# Global genetic diversity and Asian clades evolution: a phylogeographic study of *Staphylococcus aureus* sequence type 5

**DOI:** 10.1128/aac.01175-23

**Published:** 2024-01-23

**Authors:** Fengning Chen, Yuyao Yin, Hongbin Chen, Ruobing Wang, Shuyi Wang, Hui Wang

**Affiliations:** 1 Department of Clinical Laboratory, Peking University People’s Hospital, Beijing, China; Houston Methodist Academic Institute, Houston, Texas, USA

**Keywords:** methicillin-resistant *Staphylococcus aureus *(MRSA), sequence type 5, phylogenomics, pan-GWAS

## Abstract

*Staphylococcus aureus* sequence type (ST) 5 has spread worldwide; however, phylogeographic studies on the evolution of global phylogenetic and Asian clades of ST5 are lacking. This study included 368 ST5 genome sequences, including 111 newly generated sequences. Primary phylogenetic analysis suggested that there are five clades, and geographical clustering of ST5 methicillin-resistant *S. aureus* (MRSA) was linked to the acquisition of *S. aureus* pathogenicity islands (SaPIs; enterotoxin gene island) and integration of the prophage φSa3. The most recent common ancestor of global *S. aureus* ST5 dates back to the mid-1940s, coinciding with the clinical introduction of penicillin. Bayesian phylogeographic inference allowed to ancestrally trace the Asian ST5 MRSA clade to Japan, which may have spread to major cities in China and Korea in the 1990s. Based on a pan-genome-wide association study, the emergence of Asian ST5 clades was attributed to the gain of prophages, SaPIs, and plasmids, as well as the coevolution of resistance genes. Clade IV displayed greater genomic diversity than the Asian MRSA clades. Collectively, our study provides in-depth insights into the global evolution of *S. aureus* ST5 mainly in China and the United States and reveals that different *S. aureus* ST5 clades have arisen independently in different parts of the world, with limited geographic dispersal across continents.

## INTRODUCTION

*Staphylococcus aureus* is a commensal human skin and mucosal pathogen that causes infections of varying severity (1, 2). These infections can range from superficial skin infections to fatal invasive infections, such as sepsis, infective endocarditis, and osteomyelitis, which are associated with high costs of treatment and extended hospital stays (3, 4). Sequence type 5 (ST5) is a major international representative of the epidemic methicillin-resistant *S. aureus* (MRSA) clone (1) and is among the most prevalent clones causing hospital-associated infections in the western hemisphere (5, 6). ST5 has also been prevalent in Asian countries, such as China and Japan, from the 1990s to the 2020s (7–9).

Recently, ST5 has been reported as a possible predictor of bacterial persistence in adult patients with MRSA pneumonia, which may be related to ST5 strains having higher levels of vancomycin heterogeneous resistance, biofilm formation, and the presence of adhesion and virulence genes, such as *fnbB*, *tst*, and *sec* (10). In addition to high-level vancomycin heterogeneous resistance, ST5-MRSA clones have acquired multiple resistance phenotypes (11, 12), including fosfomycin resistance (10, 13). Chinese ST5 methicillin-susceptible *S. aureus* (MSSA) is recognized as a hypervirulent ST5 subtype that poses a serious clinical threat (14). Furthermore, our previous study revealed that ST5-MSSA actively expresses the *agr* system (7), which possibly accounts for its enhanced virulence. Moreover, ST5 is a major cause of animal diseases, and the majority of *S. aureus* isolates from broiler chickens are the descendants of a single human-to-poultry host jump by a subtype of the worldwide human ST5 clonal lineage unique to Poland (15).

The epidemic New York/Japan clone, ST5-SCC*mec*II, is characterized by the presence of *S. aureus* pathogenicity island (SaPIn1) (with the *tst*, *sec*, and *sel* genes) and an enterotoxin gene cluster (*egc*; with the *seg*, *sei*, *sem*, *sen*, and *seo* genes) (16), which was the predominant MRSA clone in Japanese hospitals in the 2000s (17). ST5-SCC*mec*I was the most frequent genotype among Japanese healthcare-associated MRSA strains in the early 1980s. An MRSA clone exhibiting ST5-SCC*mec*I has recently emerged in South America (18), which remains the most frequent MRSA lineage. However, this lineage is gradually being replaced by several emerging clones (19). ST5 is responsible for MRSA-associated bloodstream infections in North, Central, and South America, second only to ST8, and the diversification of ST5 was associated with independent acquisitions of unique variants of the mobile *mecA*-carrying chromosomal cassette and distinct repertoires of antimicrobial resistance genes (20). ST5-MRSA-SCC*mec*IV is now the second most common (21) community-associated MRSA clone in parts of Australia and has caused clonal outbreaks across a large geographical region (22). In China, unlike ST239, which has lost its predominant status, ST5 has spread continuously in hospital settings, according to our previous study (7, 23, 24). In general, neither the global phylogenetic relationships among the numerous ST5 clones nor the evolutionary events leading to their emergence have been determined.

In this study, we collected *S. aureus* ST5 strains, including both MRSA and MSSA, from the NCBI RefSeq database and our own strain collection. Our goals were to resolve the phylogeny, place, and time of origin of the major ST5 clones and trace the evolution of their key traits. Following phylogenetic construction and comparison of molecular characteristics among the different clades, resistance profiles and virulence gene patterns were also explored. The global population structure and phylogeography of ST5 across Asia were investigated, revealing that dominant ST5 clones from different continents have evolved independently. Furthermore, we explored the population genetic dynamics of ST5 MRSA and MSSA clades in Asia and confirmed the emergence of Asian ST5 clades to be related to the gain of prophages, SaPIs, and plasmids, as well as the coevolution of resistance genes. In summary, this work provides in-depth insights into the global evolution of *S. aureus* ST5, mainly in China and the United States, and highlights bacterial population-level differences in the emergence of Asian ST5 clades.

## RESULTS

### ST5 isolates display a tendency for geographic clustering

**Fig 1:**
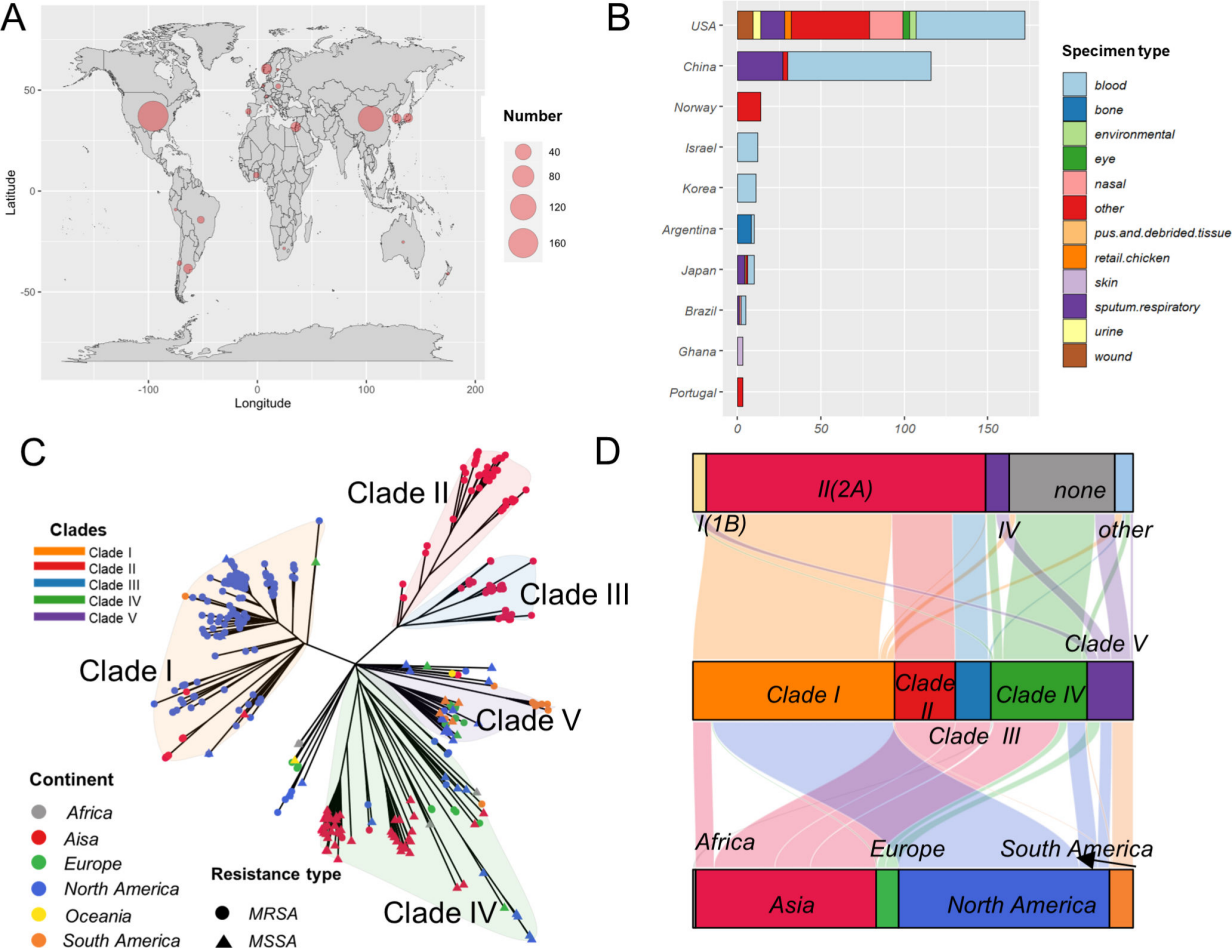
Basic information and preliminary phylogenetic analysis of *Staphylococcus aureus* ST5 isolates. (**A**) Geographic distribution of the 368 ST5 isolates. The analysis encompasses 368 isolates collected from 20 countries across Africa, Asia, Europe, North America, Oceania, and South America, with a predominant representation from China and the United States. (The map was created using the R package “maps”.) (**B**) Different specimen types from the top nine countries, with box colors distinguishing specimen types. (**C**) Tree constructed by IQ-TREE, based on the core gene alignment generated using Roary, and annotated using ggtree. Five clades were defined by resistance type, geographic distribution, and phylogenetic topology. Strain information is mapped onto the tree. MRSA strains are represented by dots and MSSA strains by triangles; tips are colored according to the continent of sampling. (**D**) Sankey diagrams for the location characteristics and SCC*mec* types of the isolates from different clades. The length of the columns represents the proportion of isolates. The thicker the line, the greater the number of isolates involved. MRSA, methicillin-resistant *Staphylococcus aureus*; MSSA, methicillin-sensitive *Staphylococcus aureus.*

**Fig 2:**
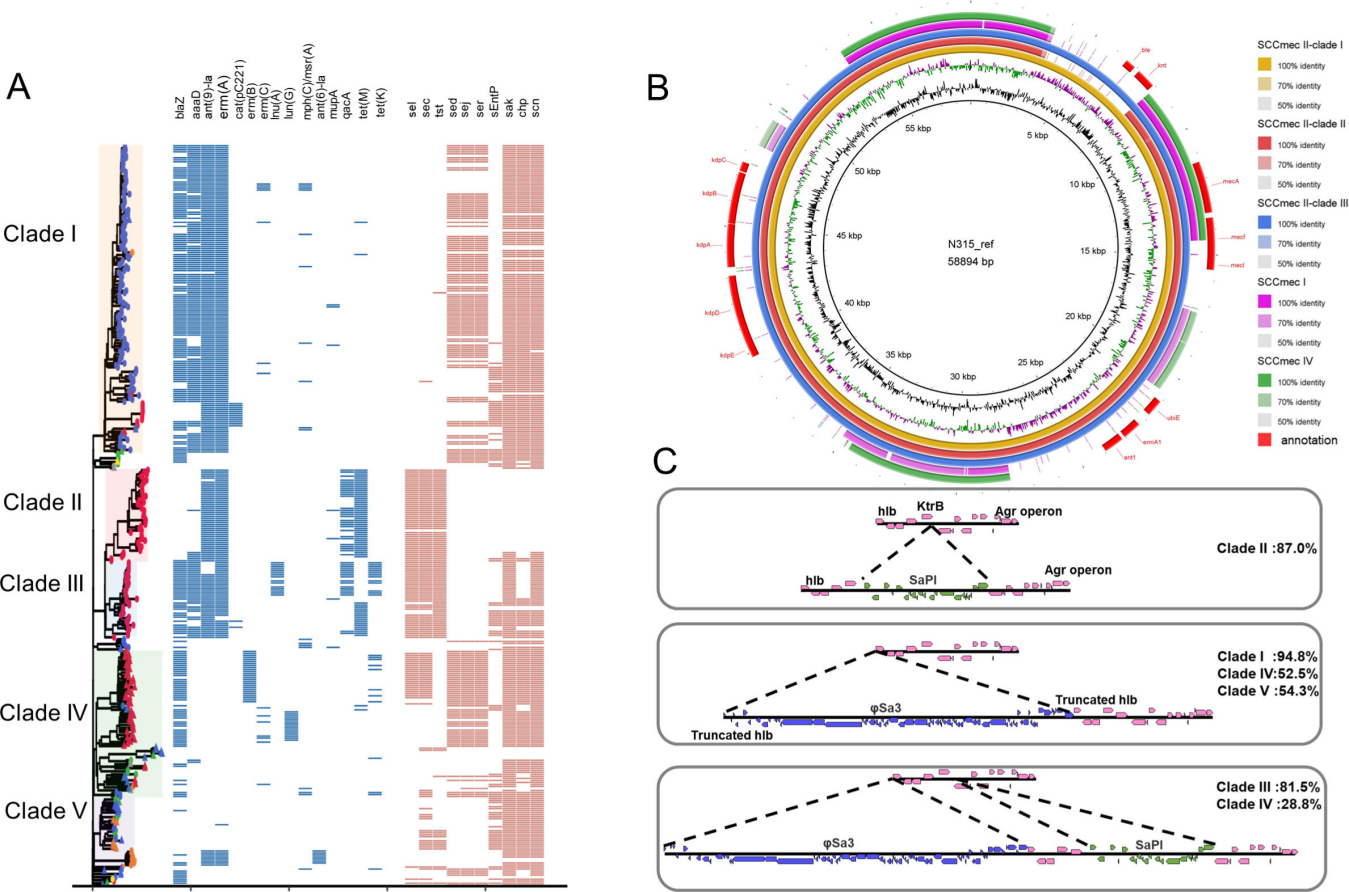
Antimicrobial resistance and virulence gene profiles of *Staphylococcus aureus* ST5 isolates. (**A**) Tree generated using R package ggtree. The five clades of *S. aureus* isolates are highlighted with colored boxes: from top to bottom are isolates in clade I-American-MRSA, clade II-Asian-MRSA, clade III-Asian-MRSA, clade IV-Asian-MSSA, and clade V, respectively. The blue panel on the right provides resistance gene characteristics of the 368 ST5 isolates based on the ResFinder database. The important virulence genes that show variations in the 368 ST5 isolates are identified in red rectangles. (**B**) Structural comparison of SCC*mec*. Comparative structural analyses of SCC*mec* from five representative isolates. The SCC*mec* in ST5 was compared with a reference SCC*mec*II sequence (*S. aureus* strain N315). Isolates are grouped in different colors, and the blank places indicate differences. (**C**) The formation of major ST5-MRSA clades is associated with the acquisition of the SaPI and prophage φSa3. The clade-specific enterotoxin genes *sel*, *sec*, and *tst* are located on the genomic island SaPI, and the immune evasion genes *sak*, *chp*, and *scn* are located on prophage φSa3. The genomic region encompassing the prophage φSa3 and enterotoxin gene island SaPI of the ST5 isolates is divided into four prevalent types according to different integrations of φSa3 and SaPI into the hotspot area. MRSA, methicillin-resistant *Staphylococcus aureus*; MSSA, methicillin-sensitive *Staphylococcus aureus.*

**Fig 3:**
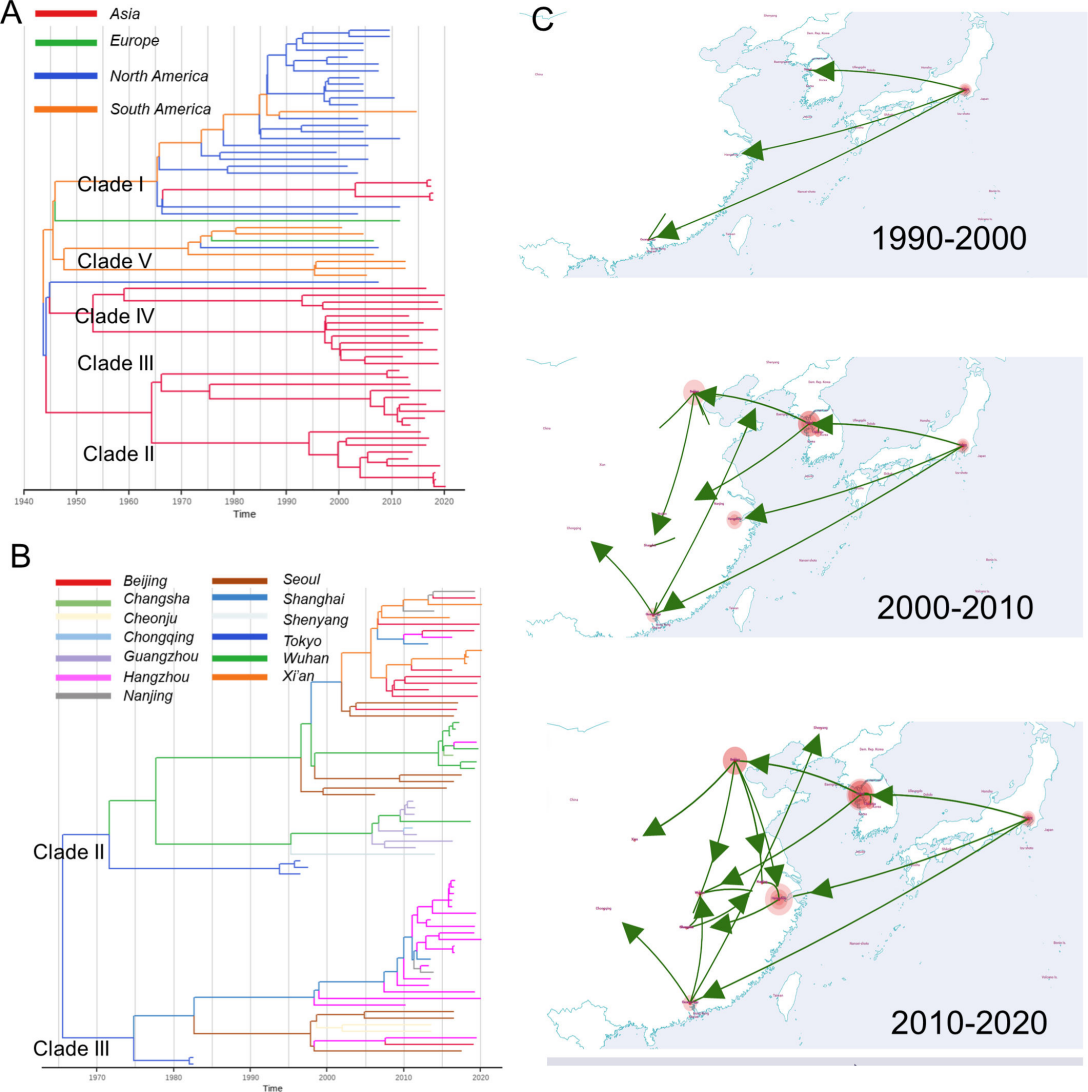
Maximum clade credibility (MCC) trees and phylogeographical inference. (**A, B**) Details of MCC phylogenetic trees of the 68 global strains and the Asian methicillin-resistant *Staphylococcus aureus* (MRSA) clades (II and III). The inferred median most recent common ancestor age of each lineage is shown with 95% highest posterior density. Terminal branches are color coded by the isolates’ continent or city of origin. Internal branches are color coded to indicate the predicted geographical origin with the highest posterior probability value inferred using maximum likelihood ancestral trait reconstruction. (**C**) Visualization of the location-annotated MCC trees for the Asian MRSA clades on the world map. The geographic spread of the Asian MRSA clades between major Asian cities was divided into three periods, that is, 1970–2000, 2000–2010, and 2010–2020. (The maps were generated using SPREAD 4 [https://spreadviz.org/].)

According to our previous analysis, *S. aureus* ST5 has remained in a stable state in China (7, 8, 25), and ST5-MSSA has emerged as a hypervirulent subclone. To investigate the characteristics of Asian *S. aureus* ST5 strains (mainly from China) in a global context, we collected publicly available whole-genome sequence data of ST5, This collection spanned 20 countries (Fig. 1A, across Africa, Asia, Europe, North America, Oceania, and South America) and 58 years (1963–2020). The most common specimen type was blood (Fig. 1B). A maximum likelihood phylogenetic tree was constructed with 257 public ST5 isolates (215 MRSA and 42 MSSA) and 111 newly sequenced Chinese ST5 isolates (44 MSSA and 67 MRSA). Five clades were defined. Clades I, IV, and V were clearly identified based on the topology of the phylogenetic tree (Fig. 1C and 2A): clade I was mainly identified as an MRSA clade and collected from North America; clade IV was identified as an MSSA clade (60/73, 82.2%) and collected from Asia; clade V comprised both MRSA and MSSA strains with diverse geographic distributions and specimen types; and clades II and III belonged to the sub-clades of the Asian MRSA clade; clade II was collected from several Chinese cities such as Beijing, Guangzhou, Wuhan, and Xi'an (Fig. 1C and 3B), whereas clade III was isolated from the southeast coastal region of China. The SCC*mec* types and geographic distribution of the clades are shown in Fig. 1D. The most prevalent SCC*mec* type was SCC*mec*II, which was present in clade I, II, and III MRSA isolates (Fig. 1D). The ST5-MRSA isolates in clades IV and V were dominated by SCC*mec*I and SCC*mec*IV, respectively.

### Antimicrobial resistance genes showed variations among ST5 clades

The presence of different resistance gene patterns and virulence gene profiles contributes to the genetic diversity among various clades. The antimicrobial resistance and virulence genes of the 368 ST5 isolates are shown in Fig. 2A. Aminoglycoside resistance genes, including *ant(6)-Ia*, *ant(9)-Ia*, and *aadD,* showed different profiles among the five clades. *AaaD* was detected in most clade I and III isolates (84.31% and 88.89%, respectively), whereas *ant(6)-Ia* was present in only seven isolates in clade V. Interestingly, *ant(9)-Ia* displayed multiple copies in a similar pattern, mainly in clade I, II, and III isolates. *TetM*, a tetracycline resistance gene, was more prevalent in clade II isolates (95.65%) than in other clades. Additionally, the chloramphenicol resistance gene *cat* (pC221) was found only in clade I isolates (7.84%). Among the lincosamide resistance genes, *lnu(A*) was present only in clade III (55.56%) and *lun(G*) in clade IV isolates (20.55%). Notably, the antiseptic resistance gene *qacA* was present only in clade II and III isolates (63.04% and 55.56%, respectively), which might provide a potential advantage for Asian ST5-MRSA (both clades II and III) to survive in hospitals, given the different disinfection practices between the United States and China. The erythromycin resistance genes *erm(B*) and *erm(C*) were mainly found in clade IV isolates (35.62% and 9.59%, respectively), and *erm(A*) displayed multiple copies in a pattern similar to that of *ant(9)-Ia*, mainly in clade I, II, and III isolates. The prevalent SCC*mec* type (SCC*mec*II) exhibited variations within the cassette region and was divided into several types with different characteristics. SCC*mec*II in clades I, II, and III, SCC*mec*I, and SCC*mec*IV were compared with a reference SCC*mec*II sequence (*S. aureus* N315, RefSeq accession number: GCF_000009645.1) using BLASTN, and the picture was generated using BLAST Ring Image Generator (BRIG). The results revealed that clade II ST5-MRSA SCC*mec*II lacked the bleomycin resistance protein-coding gene *ble* and kanamycin nucleotidyl transferase-coding gene *knt* (Fig. 2B).

### Geographic formation of major ST5-MRSA clades was associated with the acquisition of enterotoxin genes and integration of φSa3

As mentioned above, MRSA isolates were divided into clades I, II, and III. Clade II and III isolates were collected from Asia, whereas clade I isolates were collected from North America. Virulence gene analysis revealed that s*el*, *sec*, and *tst* [located on the enterotoxin island SaPI (26)] were prevalent in clade II and III isolates, whereas *sed*, *sej*, and *ser* (located on the *blaZ* plasmid) were prevalent in clades I and IV (Fig. 2A). Combined with genomic environment analysis, we found that most of the clade-specific genes were located in a hotspot area, which was flanked by the *hlb* gene (coding for β-hemolysin) and the *agr* BDCA operon. *S. aureus* harbors an Sa3int group of prophages preferentially integrated into the *hlb* gene, which encodes the human immune evasion cluster genes *sak*, *chp*, and *scn* (27). The genetic environment for φSa3 and enterotoxin islands among different clades is illustrated in Fig. 2C. Clade I isolates harbored the φSa3 prophage; clade II isolates harbored SaPI but not the φSa3 prophage; clade III isolates integrated both φSa3 and SaPI. Taken together, four typical types of combinations indicated that the geographic formation of ST5 was associated with the acquisition of SaPI and integration of φSa3.

### The phylogenetic evolution of ST5 clades was relatively independent

The regression plot (*R*^2^ = 0.358) of the full dataset of the 368 isolates inferred by TempEst was rather diffuse (Fig. S2), probably because of the uneven distribution of isolate collection dates, which may have led to a weak linear relationship and time signal. Therefore, to accomplish a relatively even distribution and infer the most recent common ancestor (MRCA) of the global clade, Bayesian inference of a timed phylogeny was applied to the 68 randomly selected genomes from the five clades, which suggested that the MRCA dated to the mid-1940s (95% highest posterior density [HPD]: 1928–1957; Fig. 3A), coinciding with the clinical introduction of penicillin. The MRCA of the Asian MSSA clade was prior to the Asian MRSA clade. Owing to the dissemination of ST5-MRSA across the Asian continent, all isolates from clades II and III were subjected to Bayesian inference of timed phylogeny. The MRCA of the Asian MRSA clade dated to the mid-1960s (Fig. 3B, 95% HPD: 1954–1974), closely preceding the use of methicillin to treat clinical infections in the early 1960s (28). Notably, the American MRSA clade (clade I) likely emerged at a time similar to that of the Asian MRSA clade (Fig. 3A). The inferred mutation rate for clade II was 1.99 × 10^−6^ (1.72 × 10^−6^ to 2.28 × 10^−6^, 95% HPD) substitutions per nucleotide site per year, which was higher than that of clade III (1.41 × 10^−6^ [1.01 × 10^−6^ to 1.81 × 10^−6^, 95% HPD]). The mutation rates for clades IV and V were 1.34 × 10^−6^ (1.05 × 10^−6^ to 1.62 × 10^−6^, 95% HPD) and 4.60 × 10^−7^ (1.50 × 10^−10^ to 9.24 × 10^−7^, 95% HPD), respectively. We failed to infer the mutation rate for clade I due to the low effective sample size (ESS) value of less than 20.

Bayesian phylogeographic inference based on ancestral state reconstruction allowed us to ancestrally trace the Asian ST5 MRSA clade to Japan (Fig. 3C), which began spreading to major cities in China and Korea, such as Guangzhou, Hangzhou, and Seoul, in the 1990s. In the period 2000–2020, descendants of Korean isolates were disseminated to Beijing and Wuhan, along with Guangzhou isolates, and spread across major Chinese cities. Interestingly, Hangzhou isolates remained restricted and showed little geographical dispersion.

### The MSSA clade displayed more genomic diversity than its MRSA counterparts

**Fig 4:**
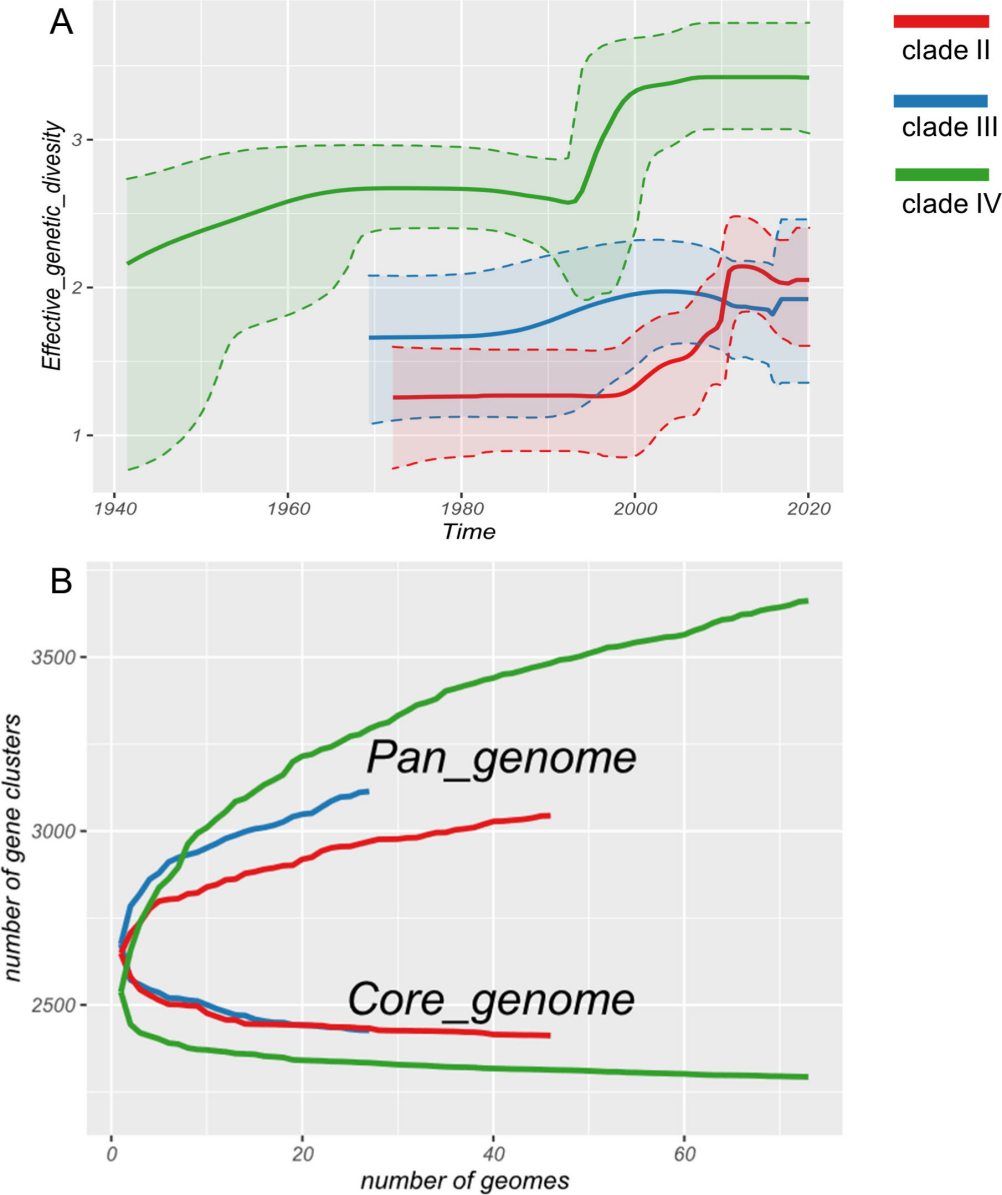
Population dynamics among the Asian *Staphylococcus aureus* isolates. (**A**) Bayesian skyline plots showing the historical changes in the effective population size of clade-II-Asian-methicillin-resistant *Staphylococcus aureus* (MRSA), clade III-Asian-MRSA, and clade IV-Asian-methicillin-sensitive *Staphylococcus aureus* (MSSA) strains. Solid lines represent the medians of estimated effective population sizes. Dashed lines and shadings indicate the upper and lower bounds of the 95% highest posterior density intervals. (**B**) Gene content diversity of clades II, III, and IV. Gene accumulation curves for the pan-genomes and core genomes (determined from the predicted coding sequences using the Roary pipeline) of the isolates from different clades.

To determine whether the MSSA clade has contributed to the persistence of ST5 in the Asian healthcare environment, a Bayesian skyline model was employed to estimate changes in the effective population size (EPS) of the Asian clades over time. EPS is estimated from the observed nucleotide variation (genetic diversity) concerning the mutation rate and can be used to infer changes in the size of a population. These models, illustrated in Fig. 4A, suggest a stable state in the EPS of clade II from the early 1970s to the early 2000s, followed by a nearly 10-fold increase over the next 10 years, which is highly consistent with previous findings that ST5 emerged in 2002 and persistently existed at a low prevalence rate (25). The EPS of clade III steadily increased from the 1980s to the 2000s, followed by a steady decrease over the next 15 years, which was exceeded by clade II in 2015. Clade IV rapidly expanded in the early 1990s and has remained steady since the early 2000s. This may partly explain why ST5-MSSA still represents nearly half of the *S. aureus* ST5 encountered nationally in the Chinese healthcare environment and could maintain the ST5 population in China (7). To verify the hypothesis that the strain-specific gene content contributes to clade formation, we performed a clade-based pan-genome analysis of the Asian clades. The isolates constituted an open pan-genome, and clade IV showed the largest pan-genome size with the addition of genomes, suggesting a frequent exchange of the gene content (Fig. 4B).

### Resistance genes played a crucial role in shaping the evolution of Asian ST5 clades

**Fig 5:**
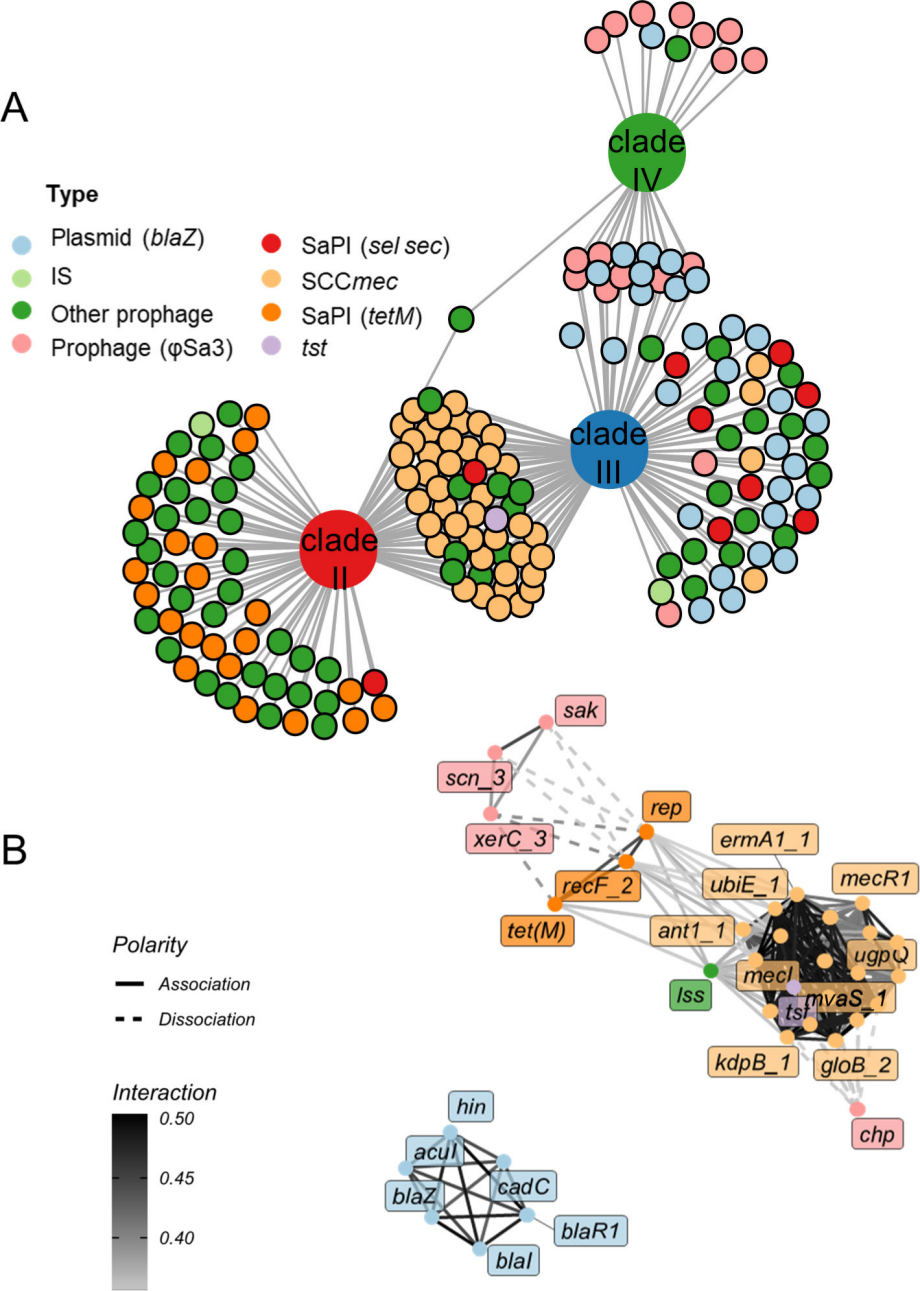
Clade-associated genes and gene co-evolution analysis. (**A**) A total of 182 genes were identified as clade-associated genes between clades II, III, and IV and visualized using the R package igraph. The Scoary algorithm was used to evaluate which gene feature is statistically associated with each clade. The cutoff for a significant association was a *P*-value lower than 1e10 and a sensitivity and specificity greater than 75%. (**B**) Gene-gene coevolution network for the significant gene pairs with scores greater than 0.35 in the full dataset and no unannotated genes, with nodes colored by gene function, edge color indicating the strength of the inferred interaction, and edge type indicating the polarity of the interaction.

A pan-genome-wide association study (GWAS) was conducted to investigate genomic differences between clades II, III, and IV. A total of 182 genes were identified as unique to one or two of the three clades. Further inspection of the genomic locations of these 182 genes suggested that mobile genetic elements, especially prophages, played an important role in the formation of the Asian clades (Fig. 5A). One hypothesis is that these genes were gained or lost in a coordinated manner to drive the formation of the Asian clades. To confirm this, the top 31 genes, which did not include unannotated genes, were subjected to gene coevolution analysis using the R package Detecting Coevolving Traits Using Relatives (DeCoTUR) (29). The two interaction clusters are shown in Fig. 5B. The largest contained the SCC*mec* cassette (*mecR*, *mecl*, *upgQ*, *kdpB*, *ermA*, *ubiE*, and *ant*) and genes involved in tetracycline resistance (*tetM*). Notably, immune evasion genes such as *sak*, *chp*, and *scn* were not associated with the presence of antibiotic genes. Another cluster primarily contained genes involved in a non-SCC*mec* resistance operon that confers beta-lactam resistance (*blaZ*, *blaI*, and *blaR1*) and genes involved in resistance to cadmium (*cadC*)—reflecting a known plasmid interaction (30). Collectively, the patterns of antibiotic resistance genes were found to have the most relevant relationships, suggesting that coevolution between resistance genes plays a crucial role in shaping the evolution of the Asian ST5 clades.

## DISCUSSION

According to our previous study, ST5 has been a rival to ST239 in Chinese hospitals since 2013 (31). Recently, ST239 lost its dominant status with its replacement by the epidemic clone ST59 (7); however, ST5 still has a place in Chinese hospitals. *S. aureus* ST5 has spread worldwide, but studies on global genetic diversity and Asian clade phylogeography of ST5 are lacking. Therefore, the study aimed to fill this gap by comprehensively investigating the phylogenetic, resistance, and virulence gene patterns, the place and time of origin of the major ST5 clones, and the evolution of their key traits.

The pathogenicity of MRSA depends, at least in part, on the toxin repertoire of each MRSA strain (32). Toxin genes are variably present in *S. aureus* isolates, possibly because they are located on prophages and genomic islands, which are thought to be mobile vectors of horizontal transfer. Importantly, this study provides strong evidence that the geographical spread of MRSA over long distances and across cultural borders is a rare event because the geographic structure of ST5 was evident at the continent, country, and even regional levels in some cases, which is consistent with a previous study (6). We found that the most remarkable difference between the Asian and MRSA strains isolated in North America and European countries was the higher carriage and coexistence rates of *tst*, *sec*, and *sel* genes in the Asian strains. In contrast, isolates encoding enterotoxin genes (*sed*, *sej*, and *ser*), which are usually found on *blaZ* plasmids (33), were prevalent in clades I and IV. This suggests that a combination of enterotoxin genes contributes to the clustering of ST5-MRSA isolates according to their geographical location.

ST5 has been successfully established worldwide, including in China, Japan, the United States, and Belgium. The ST5 New York/Japan clone is a representative worldwide clade. Asian clade III was found to have genetic traits similar to the American clade I, which belongs to the New York/Japan clone, whereas Asian clade II considerably differed from the New York/Japan clone. One explanation for the worldwide abundance of ST5-MRSA, which arises in specific geographic locations, is the repeated acquisition of SCC*mec* by ST5-MSSA strains (34). ST5 was associated with different SCC*mec* types (I, II, III, and IV). The large size of SCC*mec* type II probably limits its horizontal transfer (35); therefore, we speculate that the shorter variants of ST5-MRSA-clade II (*ble* and *knt* deletion within SCC*mec*II) have the advantage of spreading to more regions of China, along with the *tetM* and SaPI, which are specific to clade II. Interestingly, most of the ST5-MRSA-clade III isolates, which are locally endemic in China, were collected from the southeast coastal region of China, especially from Hangzhou (19/27, 70.4%). In contrast, the geographic origins of clade II were more diverse, and the isolates were collected from Beijing, Guangzhou, Wuhan, and Xi'an. Moreover, the EPS of clade II surpassed that of clade III in 2015, indicating its great potential for spreading in China.

The ST5-MSSA from China was grouped into clade IV, indicating a different genetic background from that of its MRSA counterpart. A major proportion of clade IV comprised MSSA isolates (60/73, 82.2%), and this clade had a diverse geographical origin, with isolates from Africa, Asia, Europe, North America, and South America. Therefore, it is reasonable to conclude that the EPS and gene content of clade IV are higher than those of clades II and III. Pan-genome analysis of clades II, III, and IV suggested that MGEs, such as prophages, SaPIs, and plasmids, contributed to the formation of these three clades. Further gene coevolution analysis suggested that interactions between resistance genes played a crucial role in shaping the evolution of Asian *S. aureus* ST5 clades, which rapidly acquired a suite of traits involved in antibiotic resistance, serving as a strategy to respond to antibiotic prescriptions in different regions. Moreover, prophage-associated immune evasion genes were disassociated from resistance genes, providing evidence that genetic pressures restrained the spread of resistance and virulence genes among *S. aureus* populations, thus delaying the emergence of fully virulent and resistant strains.

In summary, this is the first study to investigate the global genetic diversity and Asian clade phylogeography of *S. aureus* ST5. The findings help to gain insights into the successful epidemic of ST5 in Chinese hospitals and provide in-depth insights into the bacterial population-level differences for the emergence of Asian ST5 clades. Further studies are required to explore which role specific genetic traits play in the evolution and spread of ST5-MRSA.

## MATERIALS AND METHODS

### Bacterial isolates and culture conditions

In total, 111 *S*. *aureus* isolates, representing 44 MSSA and 67 MRSA isolates, were collected from 11 provinces and municipalities across China. We explored the phylogenetic relationships of *S. aureus* ST5 worldwide. *S. aureus* genome assemblies (*n* = 13,166) were downloaded from the NCBI RefSeq database, and ST5 assemblies (*n* = 2,652) were extracted after multilocus sequence typing (MLST) determination. To ensure genome integrity, genomes with fewer than 10 contigs were chosen for further analysis (*n* = 257). The strain selection procedure is illustrated in Fig. S1. Bacteria were routinely grown at 37°C on blood agar. Overnight cultures were grown in 5 mL of tryptic soy broth in 10-mL tubes with shaking at 200 rpm.

### Whole-genome sequencing and analysis

Whole-genome sequencing of the 111 ST5 isolates were performed on Illumina platform, and *de novo* assembly was performed using SPAdes. The assembly was annotated using Prokka 1.13.7 and the online service Rapid Annotation using Subsystem Technology (http://rast.nmpdr.org/). The core genome of *S. aureus* was determined using the pan-genome analysis pipeline Roary v3.12.2 (36). Alignments were screened for recombination using ClonalFrameML (37), and putative recombinant regions were removed before further phylogenetic analyses. Maximum likelihood phylogenetic trees were constructed using the IQ-TREE software (38). Finally, a tree was plotted and annotated using the R package ggtree (39). The MLSTs were assigned using PubMLST (https://pubmlst.org/saureus/). SCC*mec*Finder 1.2 (default threshold, 90% identity, and 60% minimum length) was used for SCC*mec* typing. Antibiotic resistance genes were detected using ResFinder (https://cge.cbs.dtu.dk/services/ResFinder/). To compare the variations within SCC*mec* in different clades, the region of SCC*mec* (nucleotide sequence from rlmH [CHECKBGO_00026] to glpE [CHECKBGO_00082]) in the reference strain N315 (GCF_000009645.1) was extracted. The BRIG software (http://brig.sourceforge.net/) was used for comparisons. Among the 368 assemblies, the virulence genes were identified using the Virulence Finder database. The hotspot genomic areas were compared using the Easyfig tool (http://mjsull.github.io/Easyfig/).

### Phylogeographic analyses

To estimate the rates of evolution and dates of the MRCAs (tree nodes), TempEst v1.5.3 (40) was used to assess whether there was a sufficient temporal signal for the phylogenetic molecular clock analysis. Because of the weak temporal signal of the 368 genomes (*R*^2^ = 0.358), 68 genomes were randomly selected from the five clades using a custom R script and subjected to model-based MRCA dating analysis using BEAST v2.6.7. Additionally, the genomes of clades II and III were selected to explore the phylogeography of ST5-MRSA isolates in Asia. ModelFinder (41) was used to determine the best-fitting substitution model, and TN93 was chosen for both alignments. Three molecular clock models (strict, relaxed log-normal, and relaxed exponential) were evaluated. Chains with 200 million generations were generated for each model combination and sampled every 20,000 generations. The first 10% of each chain was discarded as the burn-in. The convergence of the Markov chain Monte Carlo chain was inspected using Tracer v1.7.2 and through the evaluation of the ESS and parameter value traces. Only runs with ESS >200 were considered. The best-supported molecular clock model was a strict molecular clock combined with the Bayesian skyline population model. Finally, the maximum clade credibility tree was generated using TreeAnnotator v2.6.7 and plotted in ggtree.

A Bayesian discrete phylogeographic approach was used to estimate the ancestral locations of the *S. aureus* strains, and the web-based application SPREAD 4 (42) (https://spreadviz.org) was used to visualize the estimates of pathogen dispersal resulting from Bayesian phylogeographic inference on a geographic map.

### Population dynamics analysis

To investigate the population dynamics of Asian MRSA and MSSA clades, the gene content (pan-genome and core-genome) of clades II, III, and IV was determined from the predicted coding sequences using the Roary pipeline (v3.11.2) (36). A Bayesian skyline plot was constructed for clades II, III, and IV using Tracer v1.7.2 (43) to estimate changes in the EPS over time. Finally, the output results for the different clades were summarized in R v4.2.1.

### Pan-genome construction and genome-wide association analysis

Scoary was designed to highlight genes in the accessory pan-genome of a bacterial dataset associated with a particular bacterial phenotype. Here, Scoary (v1.6.16) (44) was used to establish genes typical of clade II-Asian-MRSA, clade III-Asian-MRSA, and clade IV-Asian-MSSA strains by pan-GWAS. The source of each isolate was depicted as a discrete phenotype, e.g., belonging to clade I or not, defined as “positive” or “negative,” respectively, with the Scoary algorithm evaluating which gene feature is statistically associated with each clade. The cutoff for a significant association was a *P*-value lower than 1e10 and a sensitivity and specificity greater than 75%. A total of 182 clade-associated genes were identified.

DeCoTUR was developed to detect coevolving genes in large datasets of bacterial genomes based on pairwise comparisons of closely related individuals, analogous to a pedigree study in eukaryotic populations (29). Here, DeCoTUR was used to perform gene association/dissociation analyses for the 146 *S*. *aureus* strains, including MRSA-clade II, MRSA-clade III, and MSSA-clade IV. For the pairwise distance matrix, Mashtree (v2.1) was used to calculate Mash distances (45). The clade-associated gene presence/absence matrix and distance matrix were used as inputs for DeCoTUR to obtain the coevolution scores. A total of 31 strains with no unannotated genes and scores greater than 0.35 were used to construct the gene-gene coevolution network.

## Data Availability

Sequence reads are available from NCBI Bioproject PRJNA1034752. Accession numbers for sequences are provided in Table S1.

## SUPPLEMENTAL MATERIAL

Figure S1 aac.01175-23-s0001.pdf Strain selection procedure for the 368 isolates.

Table S1 aac.01175-23-s0002.xlsx Metadata for the 368 strains.

Figure S2 aac.01175-23-s0003.pdf The regression plot of the full dataset of the 368 isolates inferred by TempEst.

ASM does not own the copyrights to Supplemental Material that may be linked to, or accessed through, an article. The authors have granted ASM a non-exclusive, world-wide license to publish the Supplemental Material files. Please contact the corresponding author directly for reuse.
