# Supplementary figures and images for: Global genetic diversity and Asian clades evolution: a phylogeographic study of Staphylococcus aureus sequence type 5
Source: Antimicrob Agents Chemother. 2024 Jan 23;68(3):e01175-23. doi: 10.1128/aac.01175-23 (PMC10916392; doi:10.1128/aac.01175-23)

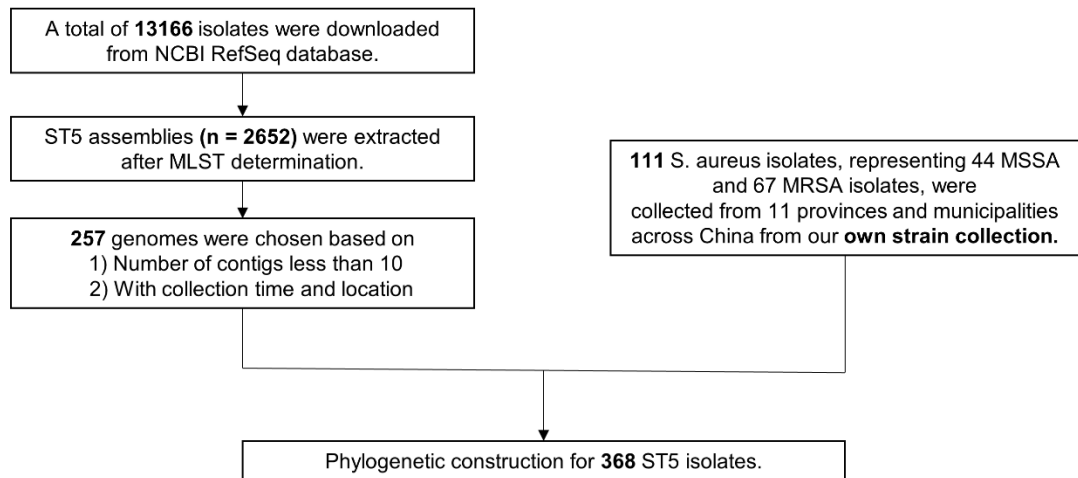

Figure S1. Strain selection procedure for the 368 isolates.

Supplement: Figure S1 — Strain selection procedure for the 368 isolates. [file aac.01175-23-s0001.pdf]

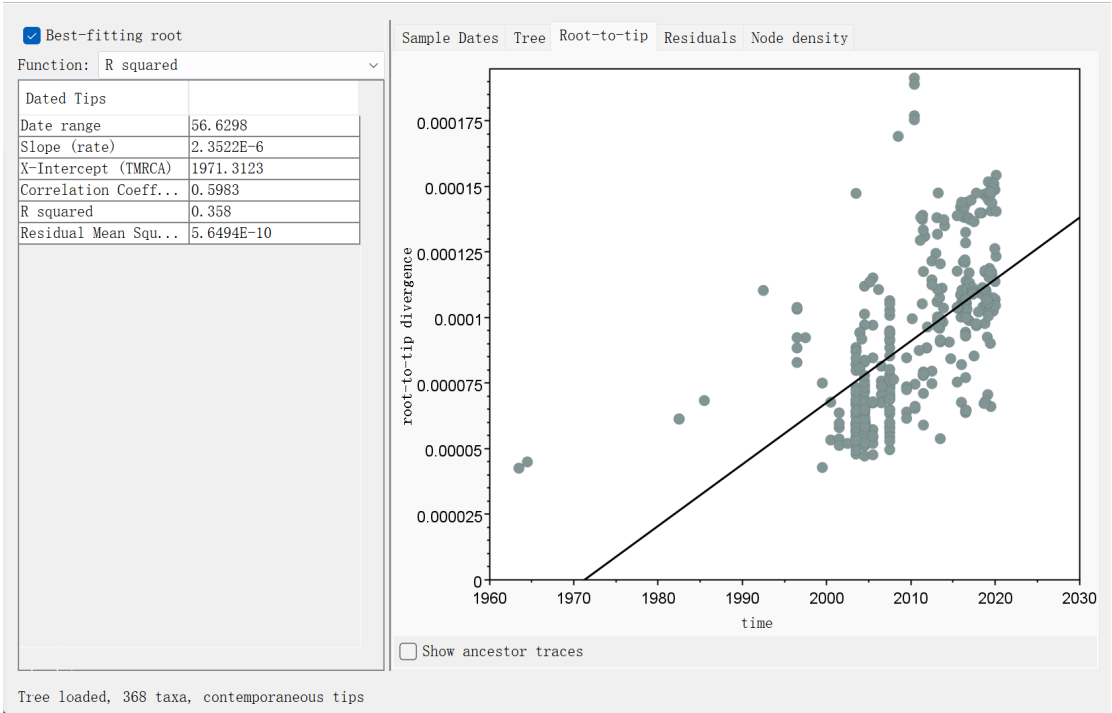

Supplement: Figure S2 — The regression plot of the full dataset of the 368 isolates inferred by TempEst. [file aac.01175-23-s0003.pdf]
